# A two-stage retrospective analysis to determine the effect of entry point on higher exit of proximal pins in lateral pinning of supracondylar humerus fracture in children

**DOI:** 10.1186/s13018-019-1400-x

**Published:** 2019-11-09

**Authors:** Xianglu Ji, Allieu Kamara, Enbo Wang, Tianjing Liu, Liwei Shi, Lianyong Li

**Affiliations:** Department of Pediatric Orthopedics, Shengjing Hospital of China Medical University, Shenyang, Liaoning Province People’s Republic of China

**Keywords:** Supracondylar humerus fracture, Pinning fixation, Proximal pin, Entry point, Exit point

## Abstract

**Background:**

Kirschner wire fixation remains to be the mainstream treatment modality in unstable or displaced supracondylar humerus fracture in children, with divergent lateral pins being the most preferred due to their sufficient stability and decreased risk of ulnar nerve injury. However, the entry point at which the proximal lateral pin can be inserted to achieve a more proximal exit and maximum divergence has not been reported. This study retrospectively analyzed the characteristics and factors influencing the entry and exit points of the proximal lateral pins.

**Methods:**

The study was divided into two stages. In stage one, the entry and exit points of the proximal pins of lateral pinning configuration were analyzed from intra-operative radiographs of children treated for extension-type supracondylar humerus fractures. The coronal and sagittal pin angles formed by the proximal pins were also measured. Using the findings of stage one, we intentionally tried to achieve a more proximal exit with the proximal pins in stage two. Comparisons between groups of patients treated by random and intentional pinnings were done statistically.

**Results:**

In the first stage, 47 (29.2%) of the 161 proximal pins exited above the metaphyseal-diaphyseal junction (MDJ) region. Of these, 85.1% entered from lateral and posterior to the ossific nucleus of the capitellum (ONC). The pin angles averaged 58.4° and 90.5° in the coronal and sagittal planes respectively. In the second stage, 47 (65.3%) proximal pins in the intended group exited above the MDJ region, while only 32 (36%) in the random group exited above the MDJ region.

**Conclusion:**

While aiming at the upper border of the distal MDJ during pinning, lateral pins can easily achieve a higher, proximal exit above the MDJ if inserted from lateral and posterior to the ONC and parallel to the humeral shaft in the sagittal plane. Higher exit can also be easily achieved in younger patients and patients fixated with smaller diameter pins.

## Introduction

Supracondylar humeral fracture (SHF) is a common circumstance in the pediatric patients. It accounts for about 60% of all pediatric elbow fractures [1]. Despite the variety in the severity of fractures and the selection of fixations, Kirschner wire fixation remains the mainstream treatment modality in unstable or displaced SHF. Generally, two or three Kirschner wires inserted from the lateral/medial and lateral condyles and configurated in crossed or divergent manner are said to provide the best stability [2–5].

Lateral entry pinning is however preferred by many surgeons because it can achieve sufficient stability while eliminating the risk of ulnar nerve injury [6–9]. The configuration of the pins should be in accordance with the fracture line to achieve the best stability. Theoretically, the pins should be divergent enough along the fracture line in order to provide the most optimum stability [10, 11]. However, the entry point at which the proximal lateral pin can be inserted for more proximal exit and therefore attain maximum divergence has not been reported yet in the literature. In some circumstances, like when the fracture lines are more oblique or located in the metaphyseal-diaphyseal junction, this configuration may be difficult to achieve, and therefore, alternative fixation techniques should be sort. In this case, knowing the limits of the exit heights of lateral pins is important for pre-operative planning. In a two-divergent lateral pinning configuration, placement of the proximal lateral pin determines the magnitude of divergence and stability of the fixation. Its limit is therefore of great clinical significance.

This study aimed at analyzing the characteristics of the entry and exit points of the proximal lateral pins and the possible influencing factors, investigating whether the position of the exit points can be intentionally controlled, and finding out how to achieve a higher exit with proximal lateral pins.

## Patients and methods

This study was approved by the Research and Ethics Committee of China Medical University. Data of patients admitted for extension-type SHF between March 2016 and December 2017 were recorded and retrospectively analyzed. The inclusion criteria were as follows: the patients were treated by closed reduction and fixated with two or more Kirschner wires by one of four attending pediatric orthopedic surgeons, who had been specialists for at least 10 years; the Kirschner wires were inserted through a lateral (radial) entry; and the patients had full sets of intra-operative fluoroscopic images in good quality. The exclusion criteria were as follows: the tip of the proximal lateral pin penetrated the anterior/posterior cortex of the humerus on the lateral view X-ray, bent pins lying within the humeri, the images were not in standard anterior-posterior (AP) and lateral views, distal fragment rotation on X-rays, and flexion-type SHF. The surgical procedure and method used to confirm reduction were done as described before [12]. The diameters of the stainless steel K-wires were 1.6 mm and 2.0 mm with pyramid tips (Double Engine Medical Material Co., Ltd., Xiamen, China).

**Fig. 1:**
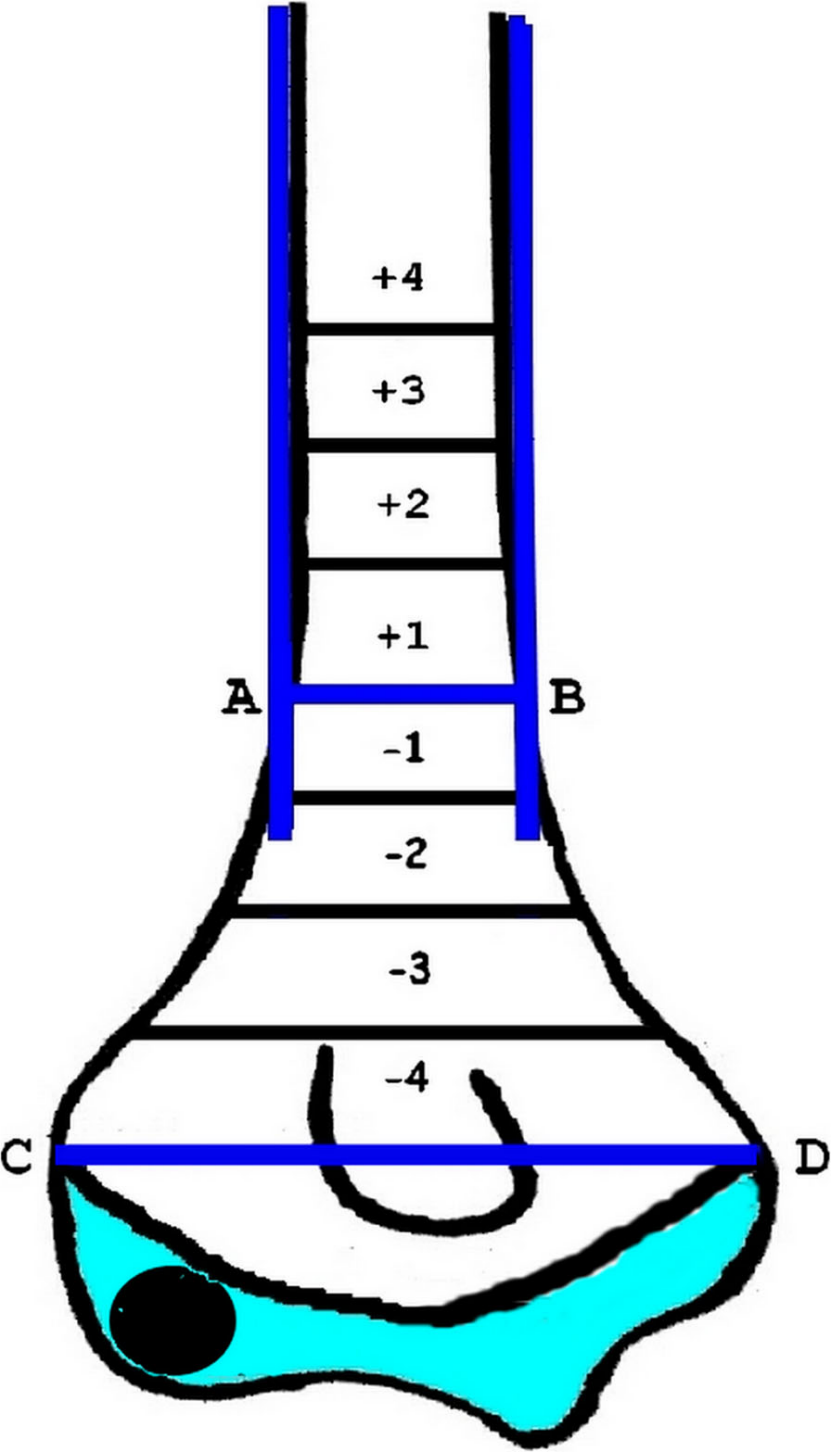
Schematic representation of the exit zones of the proximal lateral pins. Line AB represents the upper border of the metaphyseal-diaphyseal junction (MDJ) region. Line CD is the inter-epicondylar line. − 1 to − 4 are exit zones below the upper border of the MDJ; + 1 to + 4 are exit zones above the upper border of the MDJ

In the first stage of the study, data of patients treated between March 2016 and December 2016 were collected and analyzed. All the surgeons were doing the operations freely following the regulations mentioned above and standard treatment protocols. Demographic data relating to age, sex, side of injury, type of fracture, Gartland’s classification, location and pattern of fracture line, and pin size used were recorded. The last AP and lateral images before the application of casts were used for observation. Entry points of the proximal lateral pins (the most proximal lateral pin in case of more than two lateral pins) were recorded in reference to the ossific nucleus of the capitellum (ONC) on both views. To determine the exit point of the proximal lateral pin, we first determined and marked the upper border of the distal metaphyseal-diaphyseal junction (MDJ) region by drawing two perpendicular and tangential lines along the shaft of the humerus on the AP radiograph. A horizontal line (line AB) passing through the more proximal point of the two points where the two parallel lines intersected the humeral shaft was regarded as the upper border of the MDJ region (Fig. 1). The regions below and above line AB were further divided into four equal zones (− 4 to + 4) as exit zones, based on the distance from the inter-epicondylar line (line CD) to the upper border of the MDJ region (line AB) (Fig. 1).

**Fig. 2:**
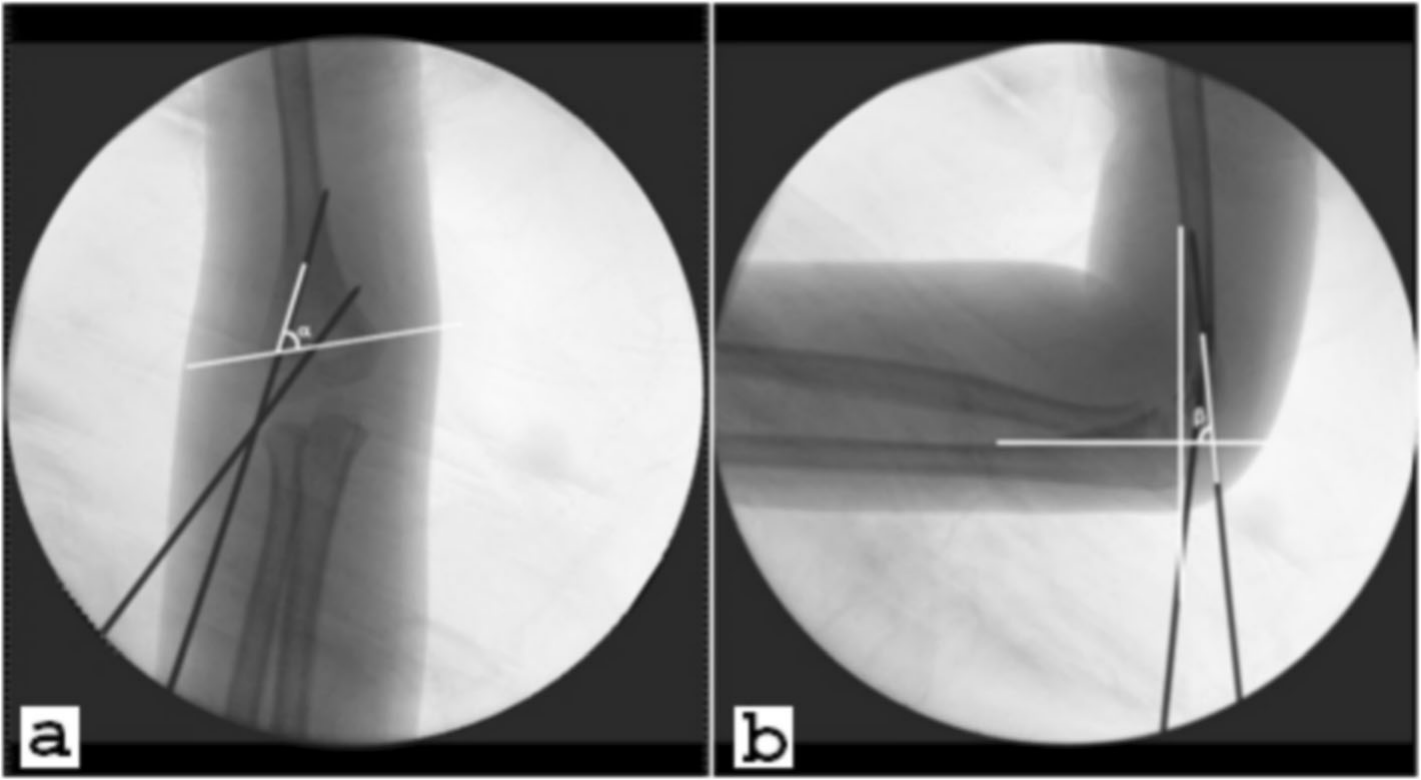
Pin angles measured on the intra-operative radiographs. **a** Coronal pin angle *α* is formed by the proximal pin and a line traversing the maximum diameter of the olecranon fossa (inter-epicondylar line). **b** Sagittal pin angle *β* is formed by the proximal pin and a line at the most distal ossified humeral bone edge, which is perpendicular to the anterior humeral line

The coronal pin angles *α*, formed by the pin and inter-epicondylar line (line CD), were measured on the AP images, while the sagittal pin angles *β*, formed by the pin and a line at the most distal ossified humeral bone edge, which was perpendicular to the anterior humeral line, were also measured on the lateral images (Fig. 2). All measurements were done using measurement tools available on the hospital’s Picture Archiving and Communication System (PACS) software (Neusoft, Shenyang, China).

**Fig. 3:**
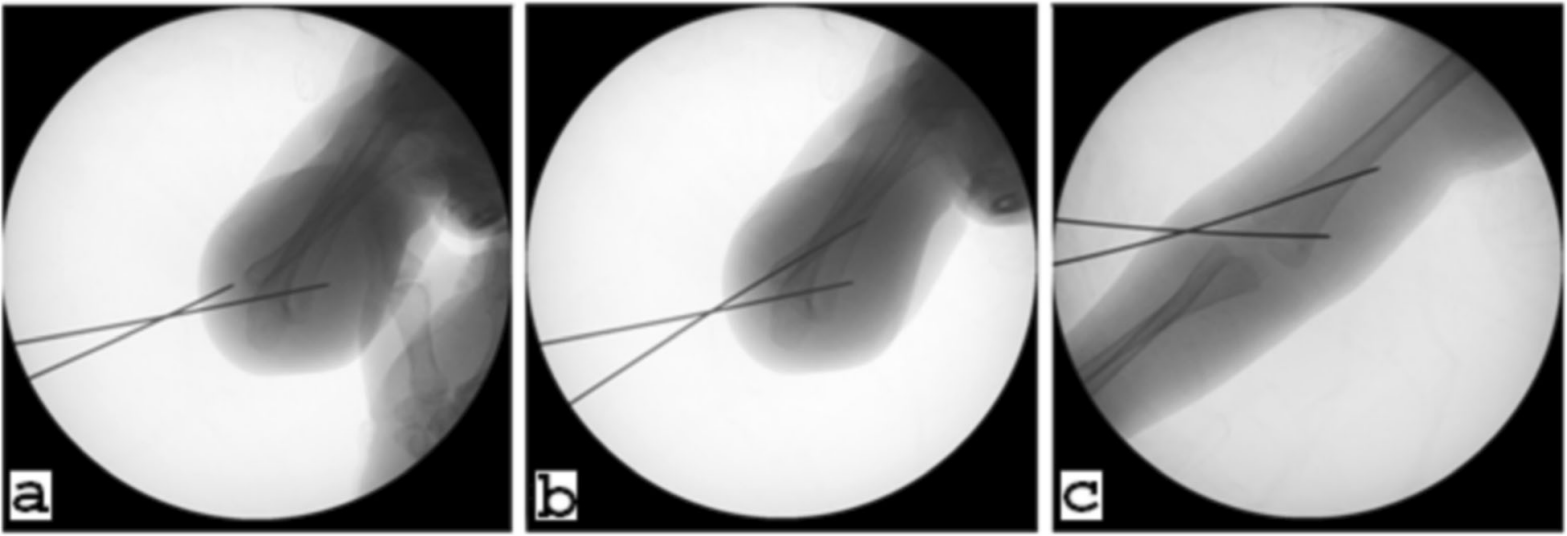
Placement of the proximal lateral pin was verified under Jones radiograph. **a** After satisfactorily placing the distal lateral pin, the proximal lateral pin was placed from the lateral distal cortex and aimed to exit in zone + 1 in the medial proximal cortex. **b** Trajectory of the proximal lateral pin within the distal humerus. **c** The proximal lateral pin exits the proximal medial cortex of the humerus

The second stage of this study started from January 2017 and ended in December 2017. Based on the findings of the first stage (see the “Results” section), two of the surgeons (EW and LS), after placing the lower/distal lateral pins, started to insert the proximal lateral pins from lateral (pins laid in the lateral third of the ONC or lateral to the ONC) and posterior (pins laid in the posterior third of the ONC or posterior to the ONC) in hyperflexed position under Jones radiographs (Fig. 3), and intentionally aimed at exiting in zone + 1. The location and configuration of the pins were confirmed by intra-operative radiographs. When the lateral pin fixation was found to be satisfactory and stable with no distal fragment rotation, removal and reinsertion of the pins for further proximal exit or insertion of another new pin was avoided. Patients were then immobilized in a long arm cast in 80 to 90° flexion for a period of 4 to 5 weeks depending on the age of the patient. This group of data was collected as the intended group, while the data of the other surgeons, who continued to fix the fractures according to the regular, standard pinning protocol, were categorized as the random group. Similar demographic data, fracture characteristics, and measurement data were also collected and recorded as in the first stage of the study.

### Data analysis

Statistical analysis of data collected was performed using SPSS version 22 for Windows (IBM Corp., New York, USA). Comparisons of categorical data were performed using the *χ*^2^ test and Fisher exact ratio tests. Continuous data were compared through independent-sample *t* test. Regression analysis was used to determine the associations between pin angles and possible influencing factors. All statistical calculations were performed with a significance level of *p* < 0.05.

## Results

### Stage I. Characteristics of the proximal lateral pins

**Fig. 4:**
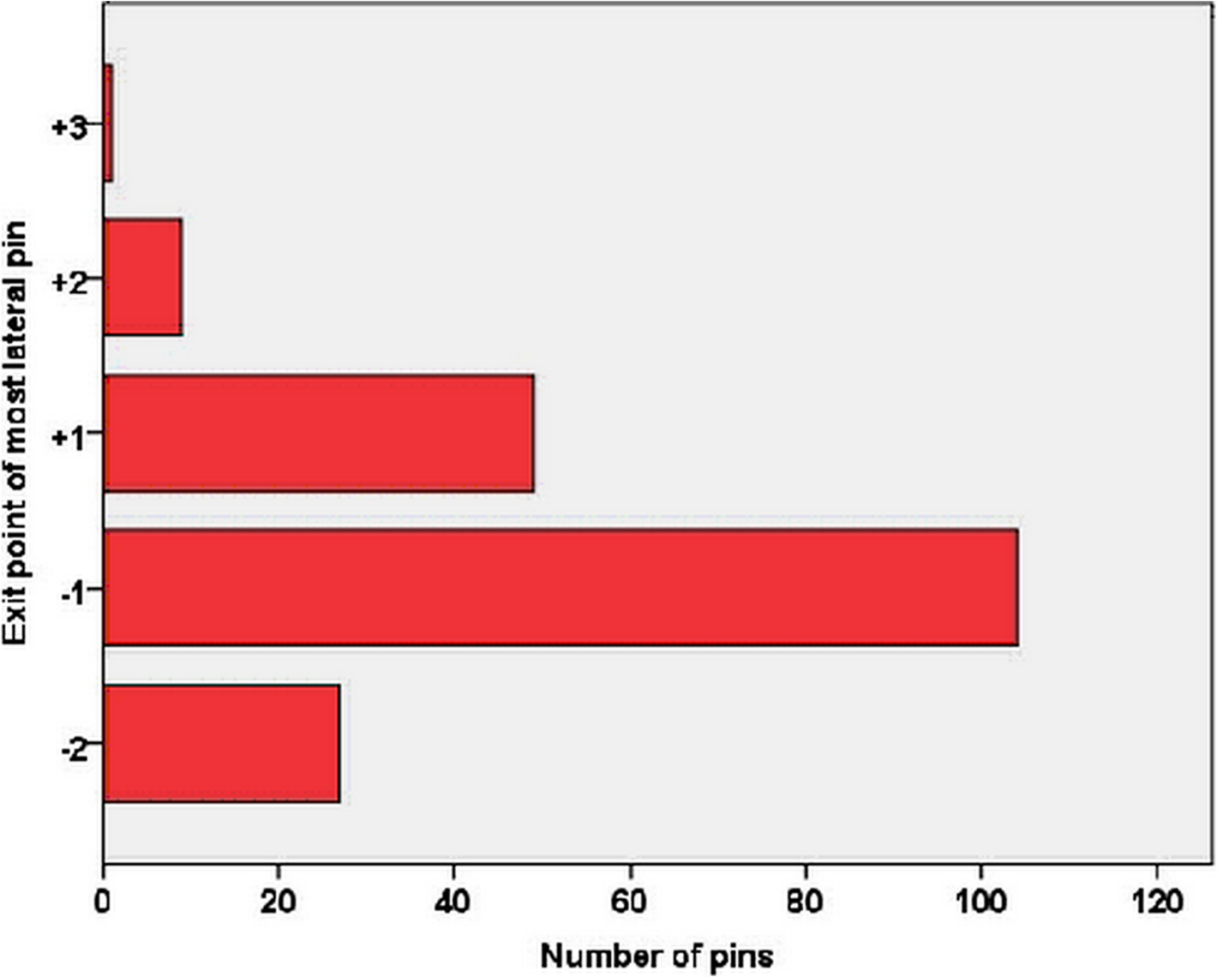
Distribution of the proximal lateral pins in the exit zones in the first stage of the retrospective study. Only 29.19% of the pins exited in zone + 1 and above

Altogether, 161 patients were included in the first stage of the study, with a mean age of 5.2 years (range 1–13 years). Demographic information and the entry and exit points of the pins in both coronal and sagittal planes are listed in Table 1. Eighty-eight (54.7%) of the pins exited in zone − 1, 26 (16.1%) in zone − 2, 38 (23.6%) in zone + 1, and 9 (5.6%) in zone + 2 (Fig. 4). The coronal pin angle averaged 58.4° (range 40.7 to 75.0°), while the sagittal pin angle averaged 90.5° (range 74.3 to 102.0°).

**Table 1:** Possible influencing factors of the exit point distribution in the first stage of the study

	Exit zones				Total	*p* value^$^
	− 2	− 1	+ 1	+ 2		
Gender						
M	17	49	16	7	89 (55.3%)	0.133
F	9	39	22	2	72 (44.7%)	
Age group						
≤ 6	18	56	31	7	112 (69.6%)	0.226
> 6	8	32	7	2	49 (30.4%)	
Side						
Right	10	32	19	2	63 (39.1%)	0.369
Left	16	56	19	7	98 (60.9%)	
Pin size						
1.6 mm	16	49	30	5	100 (62.1%)	0.098
2.0 mm	10	39	8	4	61 (37.9%)	
Gartland type						
II	6	31	12	3	52 (32.3%)	0.724
III	20	57	26	6	109 (67.7%)	
Fracture line location						
Transolecranon	23	75	37	9	144 (89.4%)	0.153
Supraolecranon	3	13	1	0	17 (10.6%)	
Fracture line pattern						
Transverse	26	80	37	9	152 (94.4%)	0.471
Medial oblique	0	3	1	0	4 (2.5%)	
Lateral oblique	0	5	0	0	5 (3.1%)	
Entry points on anterior-posterior view						
Lateral*	24	84	34	9	151 (93.8%)	0.263
Medial*	2	4	4	0	10 (6.2%)	
Entry points on lateral view						
Posterior^#^	25	75	36	7	143 (88.8%)	0.239
Anterior^#^	1	13	2	2	18 (11.2%)	
Total	26	88	38	9	161	

^$^The *p* value describes the difference in distribution of pins in the exit zones between/among subgroups (*χ*^*2*^ test)

*Lateral: proximal lateral pins laid lateral to the ONC or in the lateral third of the ONC. Medial: proximal lateral pins laid in the medial two thirds of the ONC

^#^Posterior: proximal lateral pins laid posterior to the ONC or in the posterior third of the ONC. Anterior: proximal lateral pins laid in the anterior two thirds of the ONC

We investigated the possible factors that might influence the exit height of the proximal lateral pins. Pin size and age groups made no difference on both the angles and the distribution of pins. In the coronal plane, the medial entry pins made slightly larger coronal angles than that of lateral entry pins (63.9° ± 6.43° vs. 58.0° ± 6.26°, *p* = 0.005) (Table 1). In the sagittal plane, the posterior entry pins were tilted more anteriorly than those inside the ONC (89.8° ± 5.96° vs. 96.2° ± 3.38°, *p* < 0.001). However, all the abovementioned had no significant difference with regard to the pin distribution in the exit zones (Table 1).

We also tried to find out some characteristic patterns in pins that exited relatively higher. Although the majority of the pins entered from lateral to the ONC on the AP view and posterior to it on the lateral view (85.1%), the statistical difference was insignificant (*p* = 0.308).

### Stage II. Comparison between the random and intended groups

In the second stage of the study, 161 patients also met our inclusion criteria. All the patients except 3 (1.86%) in the random group who developed a non-significant pin tract infection healed uneventfully and reported no neurovascular complications at the time of pin removal. Demographic information and pin angles for both groups are listed in Table 2. There was no difference in the constitution of the two groups except in pin size and sagittal angle of pins.

**Table 2:** General information of the random and intended groups in the second stage of the retrospective study

	Random	Intended	Total	*p* value
Age (years)	5.1 ± 3.01	4.8 ± 2.82	5.0 ± 2.92	0.532
Gender				
M	55	34	89	0.064
F	34	38	72	
Side				
Right	39	27	66	0.418
Left	50	45	95	
Gartland type				
II	20	22	42	0.245
III	69	50	119	
Fracture line location				
Transolecranon	82	66	148	0.569
Supraolecranon	7	6	13	
Fracture line pattern				
Transverse	86	70	156	
Medial oblique	1	1	2	0.913
Lateral oblique	2	1	3	
Pin size				
1.6 mm	49	64	113	< 0.001
2.0 mm	40	8	48	
Coronal pin angle *α* (°)	57.9 ± 6.70	59.7 ± 6.29	58.7 ± 6.57	0.073
Sagittal pin angle *β* (°)	90.4 ± 5.88	88.5 ± 5.59	89.5 ± 5.82	0.031

*p* value refers to the comparison between the random and intended groups (*χ*^*2*^ test or independent-sample *t* test)

**Fig. 5:**
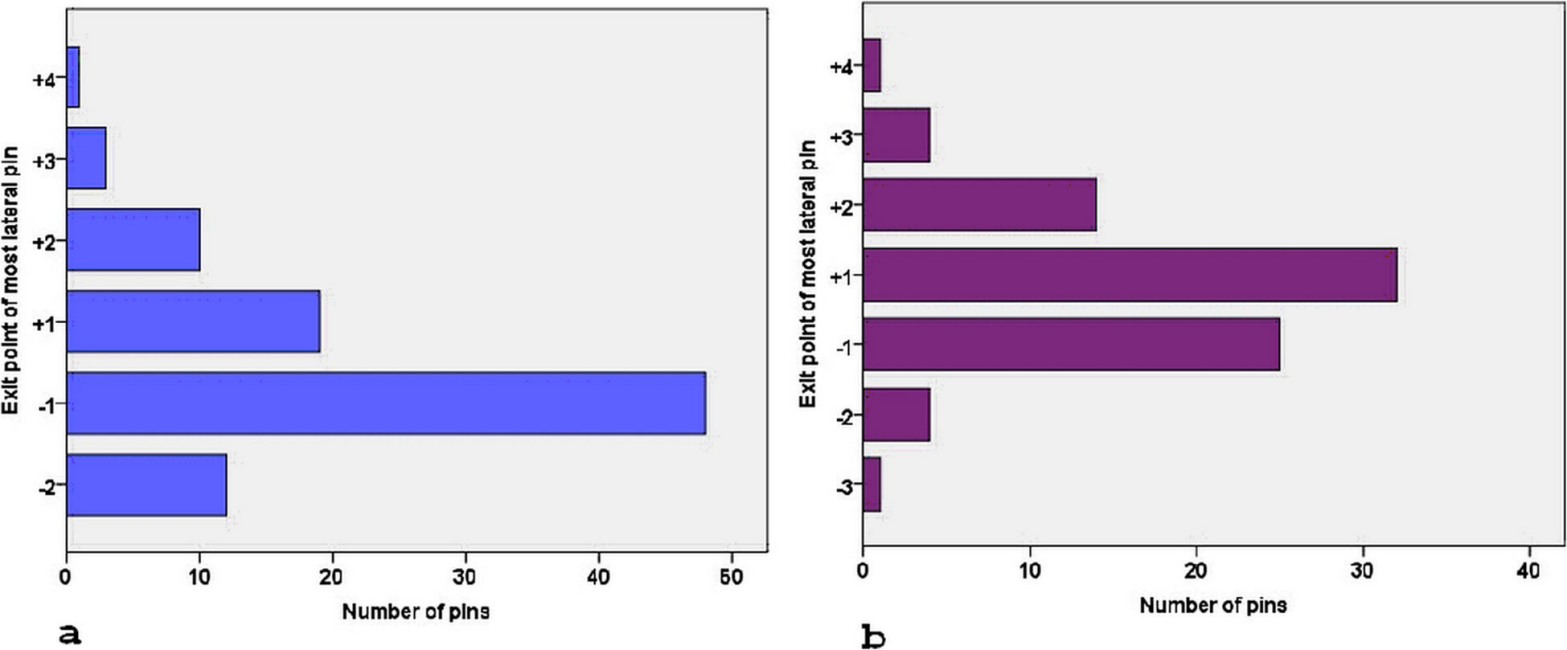
Distribution of the proximal pins in the second stage of the retrospective study **a** random group and **b** intended group. Distribution of the proximal lateral pins in the exit zones was significantly different between the two groups, *p* = 0.006

In the coronal plane, the angle of the proximal lateral pins averaged 57.9° (range 34.9 to 70.7°) in the random group, compared to 59.7° (range 46.0 to 71.6°) in the intended group. The difference in the comparison of pin angles was statistically insignificant. Distribution of the proximal lateral pins in the exit zones was significantly different between the two groups (*p* = 0.006) (Table 2, Fig. 5). In the sagittal plane, the angle of the proximal pins averaged 90.4° (range 78.5 to 109.4°) in the random group, compared to 88.5° (range 75.0 to 101.0°) in the intended group, which were significantly different (*p* = 0.031). Age group and pin size seemed to have some influence on the exit point distribution of the pins in the intended group, with *p* values of 0.046 and 0.012, respectively (Table 2).

## Discussion

The first stage of this study depicted the distribution of the proximal lateral pins used in lateral pinning of supracondylar fracture of the humerus in children. We noticed that 85% of the proximal lateral pins that exited above the MDJ region were inserted from lateral (pins laid in the lateral third of the ONC or lateral to the ONC) and posterior (pins laid in the posterior third of the ONC or posterior to the ONC). This entry point was therefore adopted for the pinning protocol in the second stage of the study. The second stage of this study demonstrated that surgeons can intentionally obtain higher exit with lateral pins when using this entry point, therefore achieving optimum divergence and greater stability.

Lateral pinning alone is a widely used pinning protocol in the treatment of unstable supracondylar humeral fractures [12–14]. Multiple studies have shown that both crossed and lateral pinning configurations are comparable in terms of stiffness. However, because crossed pins are the most implicated in iatrogenic ulnar nerve injury, some of these studies have therefore advocated for lateral pinning alone as the preferred pinning technique for SHFs, as it offers comparable stability as crossed pins and has reduced or no risk of iatrogenic ulnar nerve injury [3, 6–9]. Loss of reduction is another theoretical complication of pinning fixation method which has been attributed to many factors [10, 11, 15–18]. Though both crossed and lateral pinning configurations have been associated with loss of reduction, the incidence is a little higher with lateral pins alone [7]. For a mechanically sound fixation with lateral pins, two or three pins should be divergently constructed [13]. Choosing the right entry/starting point of the proximal lateral pin is very essential in ensuring maximum divergence and reducing loss of reduction. However, in some cases of high or oblique fracture lines, a proper divergence with bi-cortical purchase of both fragments would be somehow difficult to achieve, or even if achieved may not offer adequate stability to the fracture. In this circumstance, a clear idea about the highest exit point of lateral pins would greatly facilitate pre-operative selection of internal fixation or pin configuration and help reduce reduction loss.

By observing 161 cases fixated with lateral pinning in the first stage, the “natural” distribution of the exit points was depicted. In more than half of the cases, the proximal lateral pins exited from slightly below the MDJ line. A great majority of those that exited above the MDJ line entered from lateral and posterior to the ONC. We therefore hypothesized that the lateral and posterior region in reference to the ONC was an ideal region for inserting lateral pins when higher exit plus maximum divergence is required. The second stage of this study was therefore designed.

According to the second stage of the study, the coronal pin angles were greater in the intended group compared to the random group, indicating that the surgeons were at least partly able to direct the pins toward the intended position despite the variability in bone morphology and other factors such as swelling of the elbow and the effort to limit X-ray exposure. It might sound impractical to focus on angles in clinical practice because it was impossible to precisely pre-determine the pin angles during surgery. So we believe that using the upper borderline of the MDJ as a reference would be more applicable. Forty-seven out of the 72 pins in the intended group exited above the line, compared to 32 out of 89 in the random group. The majority of highly exiting pins in the second stage of the study were seen distributed above and below zone + 1. This demonstrates that aiming at the upper borderline of the MDJ during pin insertion would result in higher exits. In the first stage of the study, we did not find any factors that might influence the height of the exit points. In the intended group of the second stage, however, we found out that the exit points tended to be higher in younger patients and with smaller diameter (1.6 mm) pins. The bones are more soft and elastic in younger patients than in older patients, which likely made it easier for the pins to penetrate the cortex at a sharp angle. The influence of pin size, however, might also be associated with age because the 2.0-mm pins were generally used in older patients. The 2.0-mm pins were scarcely driven to the region above zone + 1 despite the effort to enlarge the coronal pin angles. Whether this was due to the more rigid bones of the patients or the characteristic of the 2.0-mm pins themselves still requires further investigation.

There were however some limitations in this study. The limited sample size in some particular groups made it hard to identify statistical significance of relevant comparisons. Only the proximal lateral pin was evaluated in this study, which we believe is the most important in determining divergence of lateral pins than the distal pin. Moreover, influence of pin numbers and configuration was not included in this study. Randomized controlled prospective studies with larger sample sizes and more standardized operative techniques are warranted to better understand these factors.

## Conclusion

This study analyzed entry points and the distribution of the exit points of proximal pins of lateral pinning alone. With intention, the exit points can be elevated to above the upper border of the MDJ. The ideal entry point to achieve that is if the pin is inserted lateral and posterior to the ONC. Higher exit point can be easily achieved in younger patients and patients fixated with smaller diameter pins. Our findings would be helpful in the pre-operative planning and selection of internal fixations in challenging supracondylar humeral fractures.

## Data Availability

The datasets in this study are available from the corresponding author on reasonable request.

## Abbreviations

AP: Anterior-posterior
MDJ: Metaphyseal-diaphyseal junction
ONC: Ossific nucleus of the capitellum
SHF: Supracondylar humerus fracture
